# g-C_3_N_4_/Ca_2_Fe_2_O_5_ heterostructures for enhanced photocatalytic degradation of organic effluents under sunlight

**DOI:** 10.1038/s41598-021-99020-6

**Published:** 2021-10-04

**Authors:** Durga Sankar Vavilapalli, Raja Gopal Peri, R. K. Sharma, U. K. Goutam, B. Muthuraaman, M. S. Ramachandra Rao, Shubra Singh

**Affiliations:** 1 Crystal Growth Centre, Anna University, Chennai, 600025 India; 2 Department of Energy, University of Madras, Chennai, 600025 India; 3 Technical Physics Division, Bhabha Atomic Research Centre, Mumbai, 400085 India; 4 Nano Functional Materials Technology Centre and Department of Physics, Indian Institute of Technology Madras, Chennai, 600036 India

**Keywords:** Chemistry, Energy science and technology, Materials science

## Abstract

g-C_3_N_4_/Ca_2_Fe_2_O_5_ heterostructures were successfully prepared by incorporating g-C_3_N_4_ into Ca_2_Fe_2_O_5_ (CFO). As prepared g-C_3_N_4_/CFO heterostructures were initially utilized to photodegrade organic effluent Methylene blue (MB) for optimization of photodegradation performance. 50% g-C_3_N_4_ content in CFO composition showed an enhanced photodegradation efficiency (~ 96%) over g-C_3_N_4_ (48.15%) and CFO (81.9%) due to mitigation of recombination of photogenerated charge carriers by Type-II heterojunction. The optimized composition of heterostructure was further tested for degradation of Bisphenol-A (BPA) under direct sunlight, exhibiting enhanced photodegradation efficiency of about 63.1% over g-C_3_N_4_ (17%) and CFO (45.1%). The photoelectrochemical studies at various potentials with and without light illumination showed significant improvement in photocurrent response for g-C_3_N_4_/Ca_2_Fe_2_O_5_ heterostructures (~ 1.9 mA) over CFO (~ 67.4 μA). These studies revealed efficient solar energy harvesting ability of g-C_3_N_4_/Ca_2_Fe_2_O_5_ heterostructures to be utilized for organic effluent treatment.

## Introduction

Energy and environmental crisis arising due to rapid industrialization, leads to organic/inorganic pollutants originating from textile, printing, polymer and pharmaceutical industries^1^. Purification of such pollutants should be carried out prior to being released into water bodies. Dyes like Methylene blue (MB), Congo red (CR) etc. and synthetic compounds like Bisphenol-A [2,2-bis (4-hydroxyphenyl) propane] or BPA are some of the widely used chemicals in industries^2–4^. Organic pollutants such as BPA etc*.* have been extensively detected in wastewater bodies, severely impact on human health^2–4^. BPA is one of the important raw materials for epoxy and polycarbonate plastics (e.g., coatings of water containers, infant bottles, and medical devices)^5^. It is extensively found in water, air and soil and acts as an endocrine-disrupting chemical. Exposure of BPA can induce carcinogenic and epigenetic modifications in humans^6^. In actual environmental conditions it’s difficult to degrade these pollutants into small non-toxic molecules^7^. Various technologies such as filtration, phytoremediation and adsorption etc., which have been adopted to treat these pollutants from wastewater, are less efficient, high cost as well consume huge energy^8–10^. Hence, it is imperative to develop clean, green and efficient methods to treat these organic pollutants by environmental friendly methods. Sunlight driven photocatalysis is one such efficient, cost-effective and environmental friendly physicochemical method to remove these pollutants from waste waters. Efficient photocatalysts are required for solar energy harvesting to treat pollutant through photocatalysis. Semiconductor based photocatalysts such as TiO_2_ and ZnO are widely used photocatalysts for water purification^11–15^. Since these materials have low photoactivity due to wider bandgap (absorbing only UV-light from solar spectrum)^16^, it is necessary to develop efficient visible light active photocatalysts for water purification. We come across some visible light active perovskite structured metal-oxide based photocatalysts such as MTiO_3_ (M: Fe, Co, Ni, Pb, Mn), LaFeO_3_, BiFeO_3_, LaCoO_3_, YFeO_3_, AgNbO_3_, NaTaO_3_, LaNiO_3,_ etc., which showed significant photocatalytic performance for degrading organic effluents^17–28^. However, the performance of such perovskite catalysts is limited by recombination of photogenerated charge carriers. The charge recombination phenomenon can be suppressed by doping with metallic or non-metallic dopants and formation of heterojunction catalysts^29^. Heterojunctions based on semiconductor composites have been reported to be effective as efficient photocatalysts by suppressing electron–hole pair recombination^30^. In this regard, Graphitic carbon nitride (g-C_3_N_4_) is an important candidate for a metal-free heterogeneous catalyst due to its robust stability and visible light responsiveness. The catalytic performance of g-C_3_N_4_ alone is unsatisfactory due to limited active sites and poor electron–hole pair separation^31–33^. Hence, a heterojunction based on metal-oxides and g-C_3_N_4_ could be an effective strategy to enhance photocatalytic performance. Brownmillerite Ca_2_Fe_2_O_5_ (CFO) is a multifunctional metal oxide which has wide range of applications in CO_2_ capturing, energy storage, fuel cells and photocatalysis etc.^34–36^. Relatively lower bandgap along with ordered oxygen vacancies in these brownmillerites is expected to be advantageous for photocatalytic applications. Oxygen vacancies in these brownmillerites can act as photoinduced charge traps to suppress the recombination of photogenerated charge carriers^34,37^. Further, the photodegradation efficiency of these compounds can be enhanced by forming heterojunction with g-C_3_N_4_. These g-C_3_N_4_/CFO heterojunctions are expected to enhance the photodegradation efficiency by efficient solar energy harvesting, improving stability and charge separation.

Therefore, in this work simple and facile synthesis of g-C_3_N_4_/CFO heterojunctions is revealed, and its morphological, structural and optical properties are investigated. The photocatalytic performance of g-C_3_N_4_/CFO heterojunctions were analyzed by degrading organic effluents MB and BPA under natural sunlight. In order to examine the photoactivity of g-C_3_N_4_/CFO composites, photoelectrochemical (PEC) studies were carried out and compared with the PEC performance of bare CFO. Systematic studies were carried out to test the performance of g-C_3_N_4_/CFO composites for solar energy harvesting applications.

## Experimental

### Material preparation

Brownmillerite nano Ca_2_Fe_2_O_5_ (CFO) was synthesized using chemical route method, as explained in previous reports^34,37^. g-C_3_N_4_ was synthesized by heat treatment of melamine in a box furnace. Melamine was first taken into a partially closed alumina crucible and then heated to 550 °C with a heating rate of 2 °C/min for 4 h followed by cooling down to room temperature^32^. The yellowish g-C_3_N_4_ mass was ground into fine powder. g-C_3_N_4_/CFO heterostructures were prepared by grinding CFO and g-C_3_N_4_ together with different contents of g-C_3_N_4_ in CFO at 10%, 25%, 50% and 75% followed by heat treatment at 300 °C. These samples were further named as CCN10, CCN25, CCN50 and CCN75 respectively.

### Characterization

Room temperature X-ray diffraction pattern of as prepared samples were recorded by Bruker D2 X-ray Diffractometer using Cu Kα radiation. The microstructure and elemental mapping of as-synthesized samples were recorded using scanning electron microscope (SEM, Jeol, 20 kV) and Energy-dispersive X-ray spectroscopy (EDS) respectively. Optical absorption spectra and concentration of effluents was analyzed using UV–visible spectroscope (Jasco V-730). The X-ray photoelectron spectra of as synthesized samples were recorded using bending magnet based Hard X-ray Photoelectron Spectroscopy Beamline at Indus-2 synchrotron source facility at RRCAT, Indore^38^.

### Photocatalytic degradation of MB and BPA

The photocatalytic performance of these heterostructures was investigated during degradation of organic effluent Methylene blue (MB) and polycarbonate plasticizer Bisphenol-A (BPA) under natural sunlight. The MB dye solution was prepared with a concentration of 1 × 10^–5^ M. 50 mg of catalyst was loaded to 100 ml of dye solution. The catalyst dye solution was ultarsonicated and placed in dark for 20 min to achieve dark adsorption equilibrium. No significant degradation of MB could be observed under dark condition. The dye catalyst solution was then placed under sunlight. At every 10 min interval the dye catalyst solution was collected and centrifuged to collect the catalyst followed by simultaneous measurement of concentration of dye solution by UV–vis absorption spectroscopy. The BPA solution with a concentration of 50 mg/L was prepared by dissolving commercially available BPA (Sigma-Aldrich, > 99%) in water. 50 mg of catalyst was loaded for 100 ml of BPA solution. The catalyst-BPA suspension was kept in sunlight and the sample was collected in regular intervals. Concentration of BPA was measured by UV–Vis absorption spectroscopy eventually. The percentage of photodegradation and first order rate constants of all samples are measured using expressions-1 and 2.

1 $$Percentage\;of\;Degradation\;(\% D) = \left[ {\frac{{C_{0} - C}}{{C_{0} }}} \right]$$

2 $$\ln \frac{{C_{0} }}{C} = kt$$

where *C*_0_ and *C* are the concentrations of effluent at 0 min and at corresponding time interval respectively. k is the degradation rate constant.

### Photoelectrochemical (PEC) studies

Photoelectrochemical (PEC) performance of CFO and g-C_3_N_4_/CFO composite was investigated using Electrochemical workstation (Autolab, PGSTAT 204 FRA32M) under illumination of 100 mW/cm^2^ (1 Sun) of light intensity. Photoelectrodes are prepared by coating slurry of active material on FTO (a mixture of α-terpineol and ethyl cellulose mixture used as binder).

## Results and discussion

**Figure 1:**
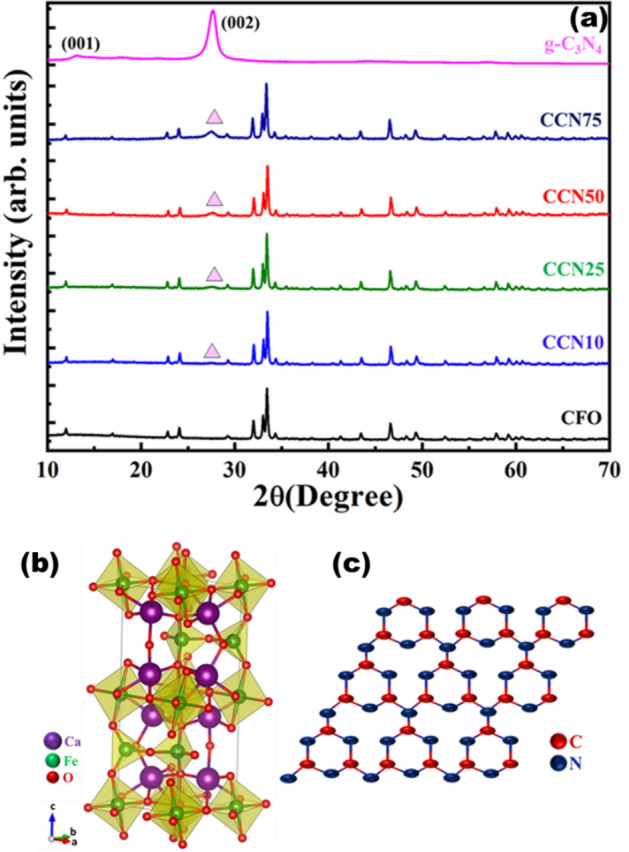
(**a**) XRD pattern of the CFO, g-C_3_N_4_, CCN10, CCN25, CCN50 and CCN75. (**b** and **c**) Structures of brownmillerite Ca_2_Fe_2_O_5_ and layered g-C_3_N_4_ respectively.

Structural phase analysis of Ca_2_Fe_2_O_5_, g-C_3_N_4_ and g-C_3_N_4_/CFO composites was carried out by powder XRD as shown in Fig. 1(a). The XRD pattern of CFO is in good agreement with orthorhombic crystal system (Fig. 1(b))^35^. XRD pattern of g-C_3_N_4_ consists of two diffraction peaks at 13.1° and 26.7° which correspond to characteristic lattice planes (001) and (002) respectively^39^. The stacked 2-dimensional graphite like structure of g-C_3_N_4_ is shown in Fig. 1(c). With increase in g-C_3_N_4_ content in g-C_3_N_4_/CFO composite from 10 to 75%, the intensity of diffraction peaks corresponding to g-C_3_N_4_ increased gradually without further secondary phase formation.

**Figure 2:**
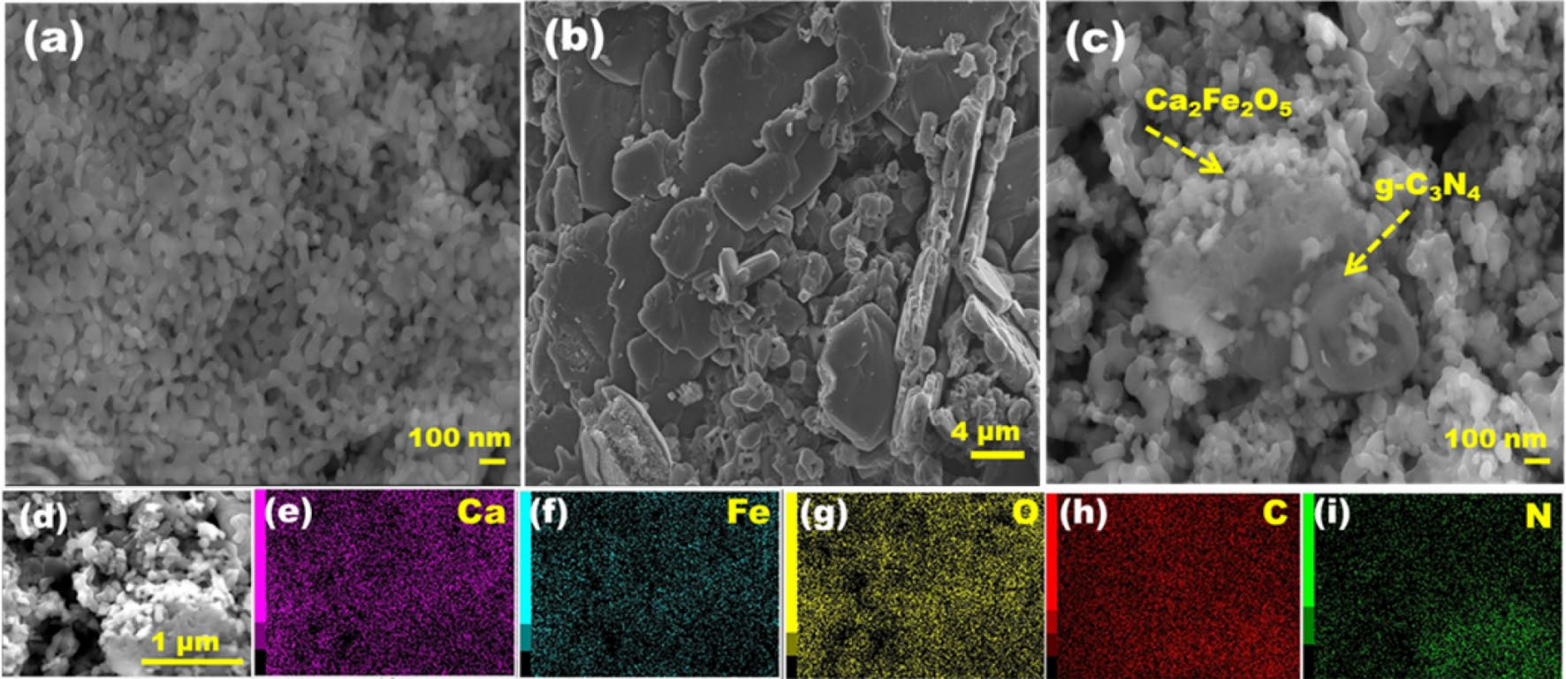
SEM images corresponding to (**a-c**) CFO, g-C_3_N_4_ and CCN50 (**d–i**) EDS mapping images of CCN50 sample showing uniform distribution of Ca, Fe, O, C and N elements.

Morphology of CFO, g-C_3_N_4_ and g-C_3_N_4_/CFO composite (CCN50) samples are shown in Fig. 2(a-c). EDS analysis was carried out to investigate the distribution of constituent elements in composite CCN50. From elemental mapping, uniform distribution of Ca, Fe, O, C and N elements were observed throughout the sample (Fig. 2(d-i)).

**Figure 3:**
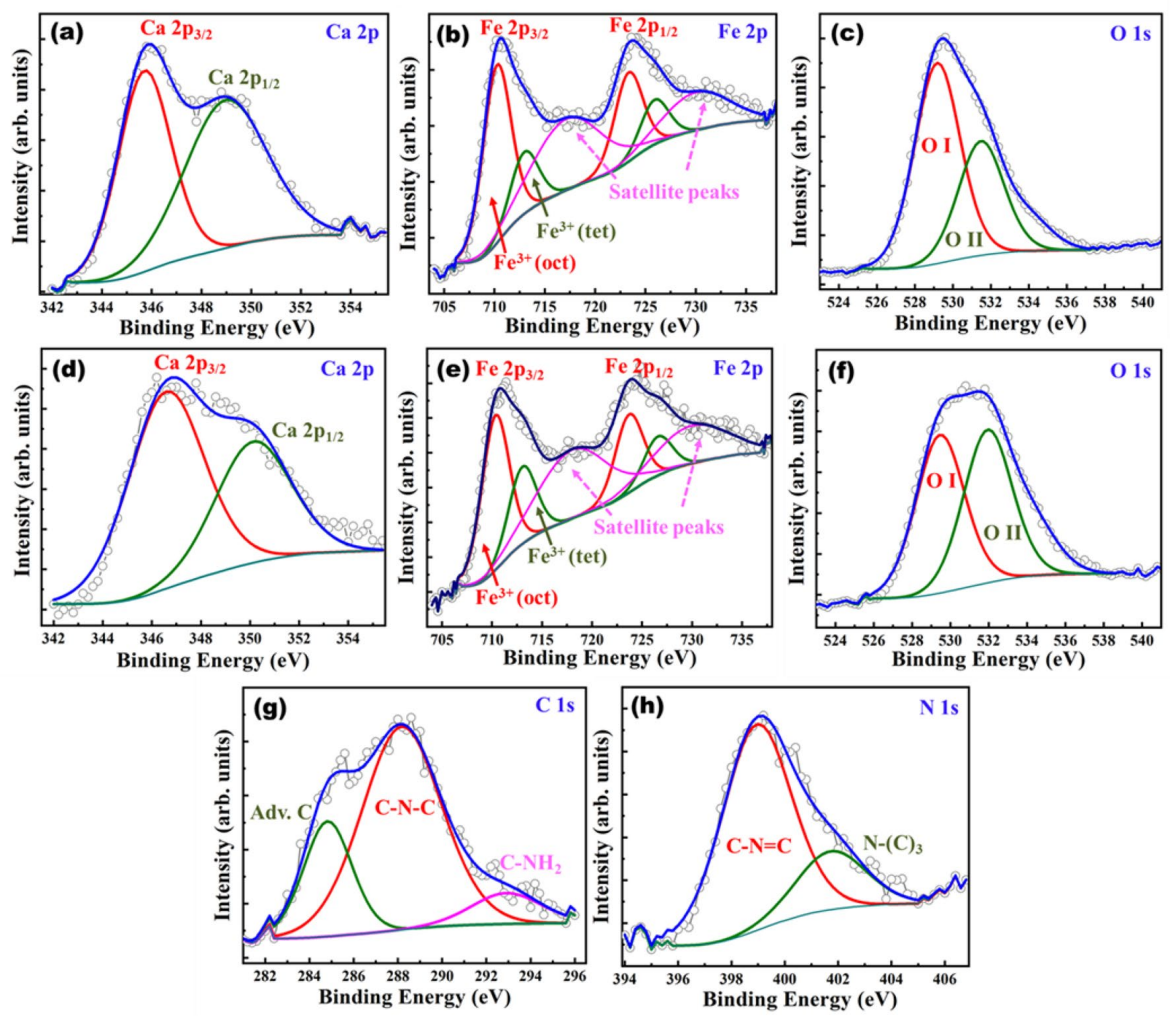
X-ray photoelectron spectra (de-convoluted) and the corresponding fits corresponding to CFO and CCN50 samples: in CFO (**a**) Ca 2p, (**b**) Fe 2p (**c**) O 1 s and, in CCN50 (**d**) Ca 2p, (**e**) Fe 2p, (**f**) O 1 s, (**g**) C 1 s, (**h**) N 1 s.

The chemical composition and the oxidation states of constituent elements were analyzed using XPS. XPS measurements of CFO and CCN50 samples were shown in Fig. 3(a-h). XPS spectra of Ca 2p of CFO and CCN50 shown in Fig. 3(a&d) constitute of two peaks arising from spin orbit coupling of Ca 2p_3/2_ and Ca 2p_1/2_. In CFO, Ca 2p_3/2_ and Ca 2p_1/2_ peaks appeared at ~ 345.7 eV and ~ 349 eV respectively with a difference of ~ 3.3 eV. For CCN50 samples, Ca 2p spin orbit splits into two peaks and lies at ~ 346.5 eV and ~ 350.1 eV with a binding energy difference of ~ 3.5 eV. This implies that, in both CFO and CCN50 samples Ca existed in 2+ oxidation state^35^. The XPS spectra of Fe 2p for CFO and CCN50 are shown in Fig. 3(b&e). The peaks arising due to spin orbits split namely Fe 2p_3/2_ and Fe 2p_1/2_, are further deconvoluted into two peaks, corresponding to octahedral (FeO_6_) and tetrahedral (FeO_4_) coordination peaks of Fe^40,41^. Brownmillerite Ca_2_Fe_2_O_5_ consists of alternative layers of FeO_6_ octahedra and FeO_4_ tetrahedra as shown in Fig. 2a. In CFO sample, Fe 2p_3/2_ and Fe 2p_1/2_ peaks are appearing at 710.6 eV and 723.7 eV respectively with a binding energy difference ~ 13.1 eV. A characteristic satellite peak corresponding to Fe is also observed at a difference of about 7.1 eV from Fe 2p peaks. For CCN50 sample Fe 2p_3/2_ and Fe 2p_1/2_ peaks appeared at 710.7 eV and 723.9 eV respectively with a B.E difference ~ 13.2 eV. This indicates that in both samples Fe existed in 3+ oxidation state^41,42^.The presence of octahedra and tetrahedra coordination species of Fe in Fe 2p XPS spectra is a clear indication of oxygen vacancies in brownmillerite CFO and CCN50 samples^35,43^. The XPS spectra corresponding to O1s for both CFO and CCN50 samples were deconvoluted into two peaks [Fig. 3(c&f)] and named as OI and OII. The OI peak corresponding to lattice oxygen appears at 529.2 and 529.5 eV for CFO and CCN50 samples respectively. OII peaks appear at 531.5 and 531.8 eV respectively for both samples and is attributed to chemisorbed oxygen species^42^. The XPS spectra corresponding to C 1s and N 1s for CCN50 sample is shown in Fig. 3(g&h). For C 1s, XPS spectra was deconvoluted into three peaks located at 284.8 eV, 288.1 eV and 292.9 eV. The peak at 284.8 eV corresponds to surface adventitious carbon. The peaks at 288.1 eV and 292.9 eV are attributed to sp2 hybridized (C-N–C) bond present in g-C_3_N_4_ aromatic ring and the C-NH_2_ bond respectively^44–46^. In case of N 1s, the XPS spectra could be deconvoluted into two peaks located at 398.9 eV and 401.7 eV. These two peaks can be attributed to sp2 hybridized (C-N = C) bond in the trizine rings and the N atoms in the ternary N-(C)_3_^46^. These results confirm the presence of sp2-bonded g-C_3_N_4_ in CFO and formation of g-C_3_N_4_/CFO heterostructure.

**Figure 4:**
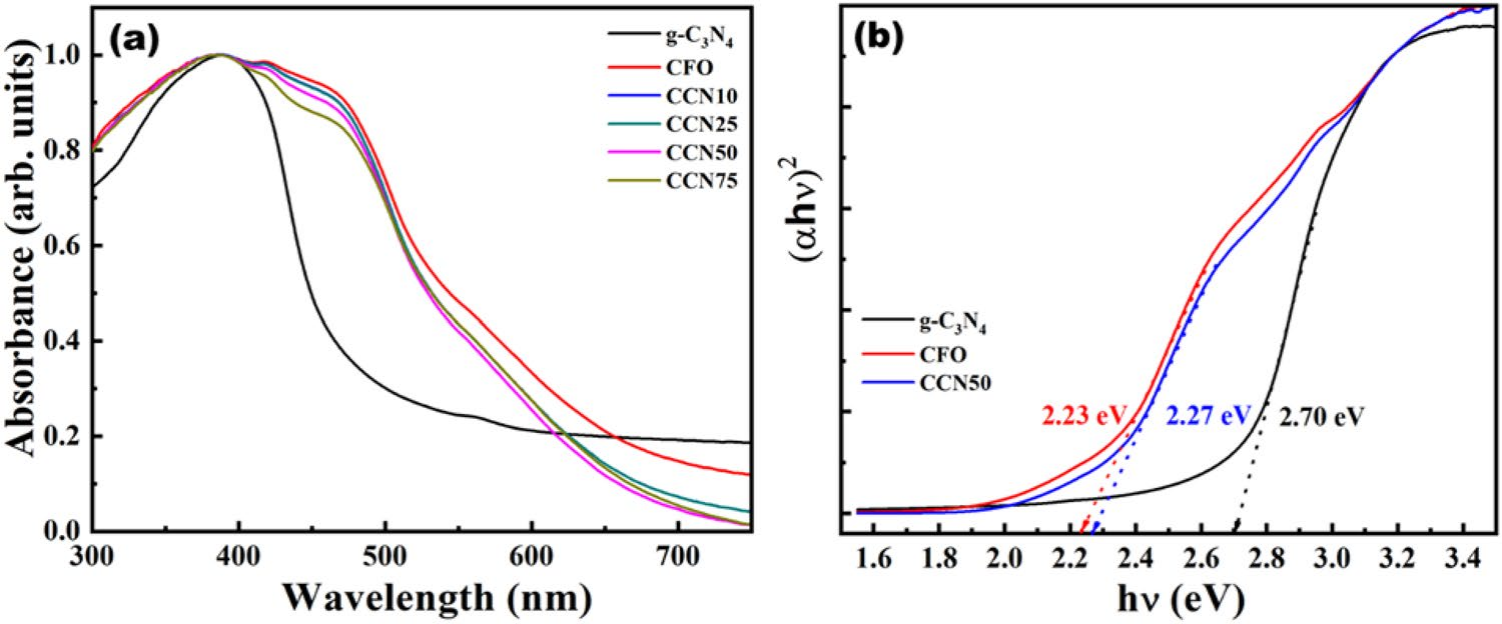
(**a**) UV–vis absorption spectroscopy of CFO, g-C_3_N_4_, CCN10, CCN25, CCN50 and CCN75 samples (**b**) Tauc plots corresponding to CFO, g-C_3_N_4_ and CCN50.

The optical properties of g-C_3_N_4_/CFO heterostructures were studied using UV–visible absorbance spectroscopy. Optical absorbance of CFO, g-C_3_N_4_ and g-C_3_N_4_/CFO heterostructures are shown in Fig. 4a. The absorbance of as synthesized heterostructures appears entirely in visible region and is highly desired for sunlight driven photocatalysis. The corresponding bandgap of CFO, g-C_3_N_4_ and CCN50 obtained from Tauc plot is shown in Fig. 4b. The bandgap of g-C_3_N_4_, CFO and CCN50 are 2.7 eV, 2.23 eV and 2.27 eV respectively. Preliminary sunlight-driven catalytic activity of optimized g-C_3_N_4_/CFO heterostructures was investigated through photodegradation of MB and BPA.

In order to enhance the photocatalytic activity over bare CFO and g-C_3_N_4_, g-C_3_N_4_/CFO heterostructures were synthesized to reduce the recombination of photogenerated electron–hole pairs and effective charge separation. g-C_3_N_4_ has a 2-dimentional graphite like structure constituting of π-conjugated systems, which are responsible for delocalization of electrons throughout the π-network^47^. The poor photocatalytic activity of g-C_3_N_4_ is attributed to the recombination effects of photogenerated electron (e^-^)–hole (h^+^) pairs due to coulombic forces^48^. Hence the optimum way to enhance the photocatalytic activity is through formation of composite or heterojunction of g-C_3_N_4_ with other metal-oxides.

**Figure 5:**
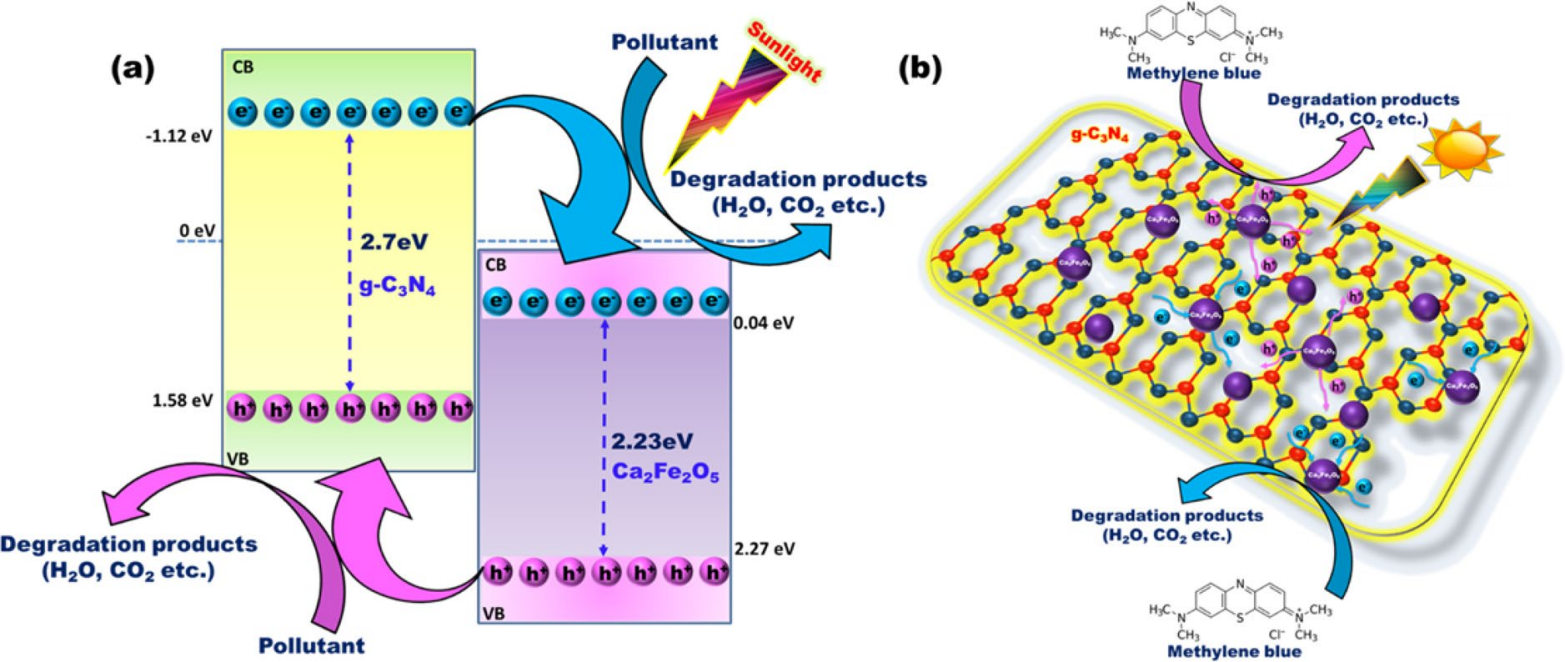
Schematic diagram of (**a**) Type-II heterostructure of g-C_3_N_4_/CFO (**b**) photocatalytic degradation mechanism of MB using g-C_3_N_4_/CFO heterostructure.

The photocatalytic degradation mechanism of CFO/g-C_3_N_4_ heterostructures [Fig. 5(a&b)] is evaluated based on the energy band positions of valence band (VB) maxima and conduction band (CB) minima. The VB maximum and CB minimum positions of g-C_3_N_4_ lie at ~ 1.58 eV and ~ -1.12 eV respectively, whereas for CFO they lie at 2.27 eV and 0.04 eV respectively, forming a type-II heterojunction between CFO and g-C_3_N_4_ [Fig. 5a]. This type-II heterojunction helps to mitigate the e^-^-h^+^ pair recombination, thus improving the photocatalytic process. Individual optimization of photodegradation efficiency by CFO and g-C_3_N_4_ samples provided a pathway to synthesize and investigate the g-C_3_N_4_/CFO composites. The g-C_3_N_4_/CFO composites are then used for degrading BPA under direct sunlight.

**Figure 6:**
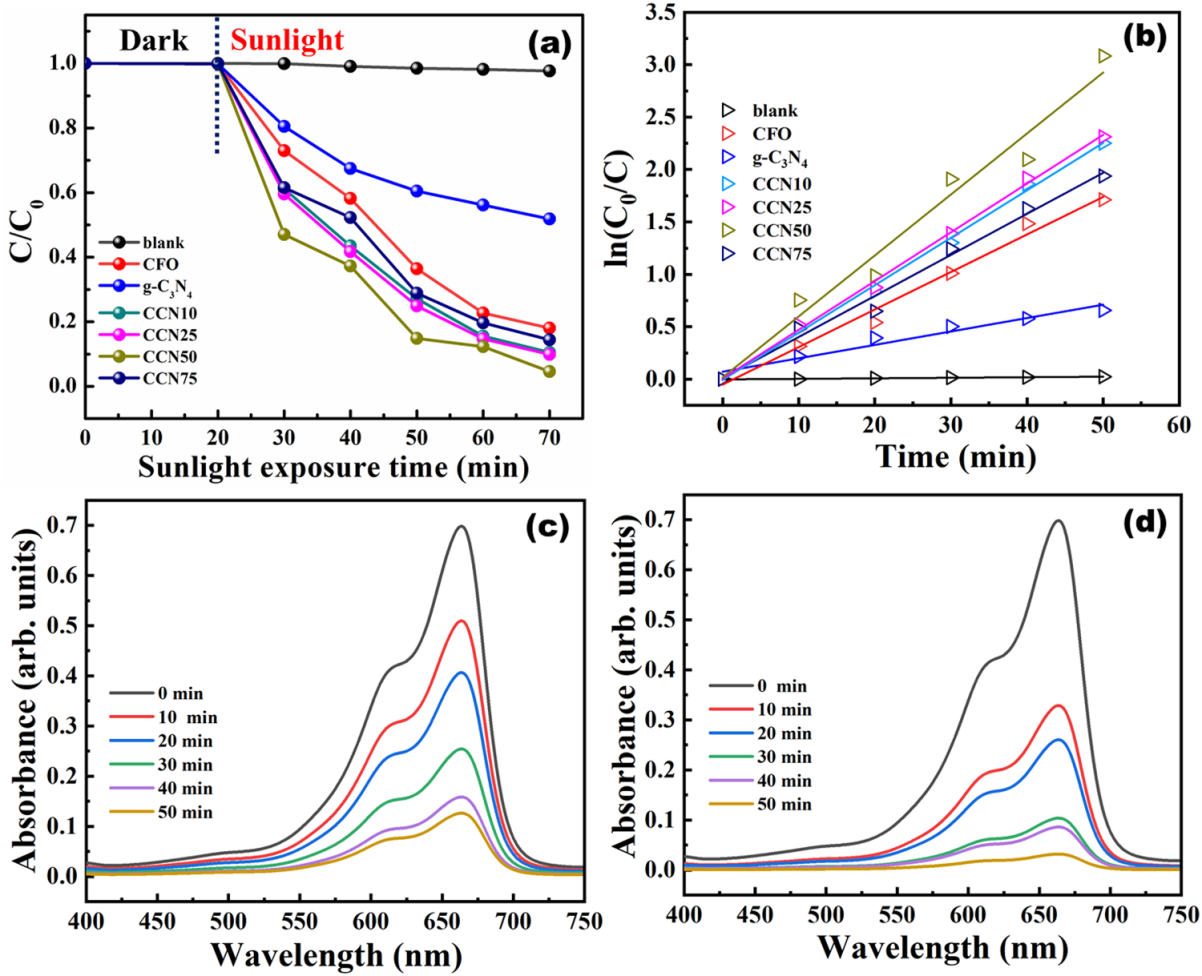
(**a**) Photocatalytic performance of activity of CFO, g-C_3_N_4_, CCN10, CCN25, CCN50 and CCN75 to degrade MB (**b**) corresponding reaction kinetics, and photodegradation profile of MB using (**c**) CFO (**d**) CCN50. (reaction conditions: pH = 7.2, temperature = 32 °C).

The percentage of degradation, first order rate constant plots and degradation profile of MB using CFO and CCN50 are shown in Fig. 6(a-d). Among the as synthesized samples, CCN50 showed higher photodegradation efficiency of about 96% with a rate constant 0.058 min^-1^. This was found to be much higher than the photodegradation efficiencies of bare CFO and g-C_3_N_4_ which showed photodegradation efficiencies of about 81.9% (0.035 min^-1^) and 48.1% (0.013 min^-1^) respectively. These studies imply that 50% of g-C_3_N_4_ in CFO provides an optimal composition to form efficient heterostructure photocatalysts. An excessive g-C_3_N_4_ content in g-C_3_N_4_/CFO could cover the surface of CFO, which reduces the photon absorption of heterostructure and also the formation of heterojunction between g-C_3_N_4_ and CFO could be suppressed by the inclusion of excessive g-C_3_N_4_ due to aggregation phenomenon^49^. Less quantity of g-C_3_N_4_ is a problem for efficient photodegradation, which may not suppress electron–hole recombination efficiently, hence the optimum content of g-C_3_N_4_ loading plays a vital role in photodegradation performance of heterostructures. In this article 50% of g-C_3_N_4_ content in CFO was found to be an optimal composition for efficient photodegradation.

**Figure 7:**
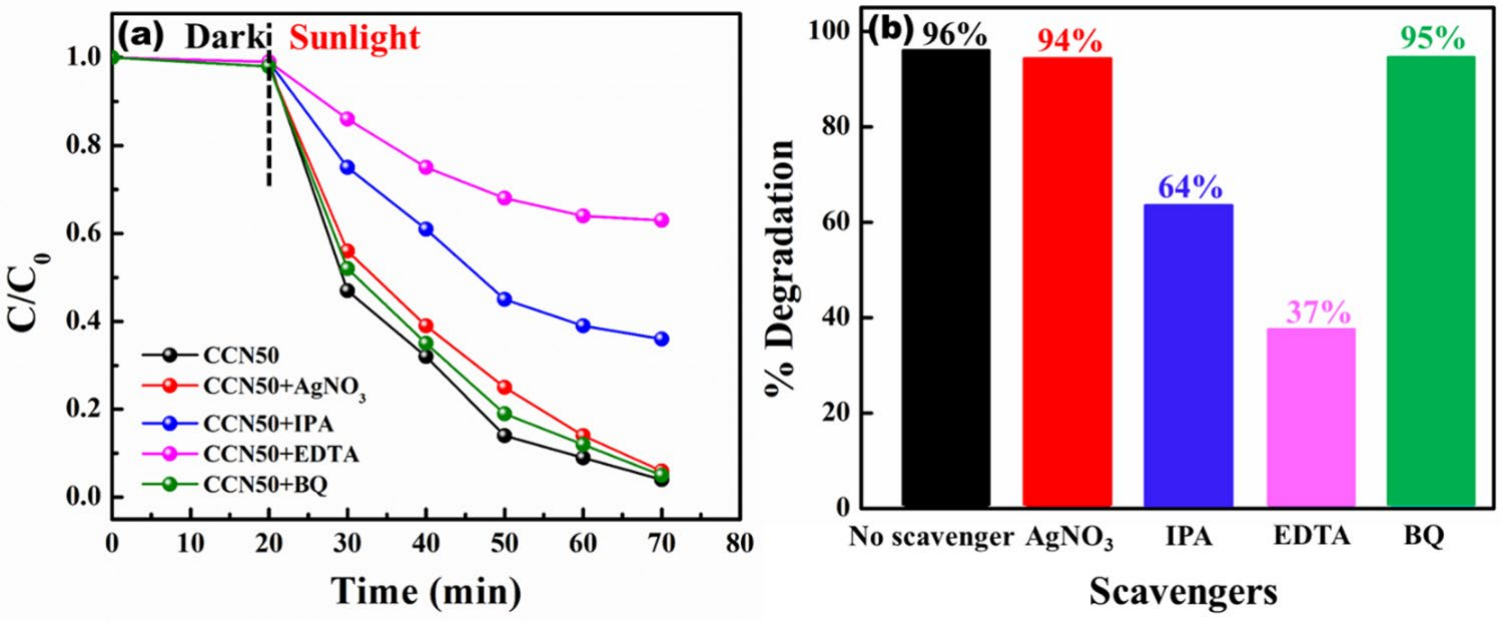
Comparisons of photocatalytic activities of CCN50 for the degradation of MB with and without various scavengers (**a**) C/C_0_ plots (**b**) percentage of degradation.

The photocatalytic mechanism of g-C_3_N_4_/Ca_2_Fe_2_O_5_ (CCN50) heterostructure to degrade MB was explained in detail using active species trapping experiments (Fig. 7(a&b)). Various scavengers such as AgNO_3_ (1 mmol), isopropyl alcohol (IPA, 1 mmol), ethylenediaminetetraacetic acid (EDTA, 1 mmol), and benzoquinone (BQ, 1 mmol) are taken as electron (e^-^), hydroxyl radicals (•OH), holes (h^+^), and superoxide radicals (O_2_^-^) trapping agents respectively. By adding electrons and superoxide radical trapping agents, the degradation of MB doesn’t change much but the photodegradation efficiency rapidly decreased to 64% and 37% from 96% by adding holes and hydroxyl radical trapping agents respectively. This implies that the photocatalytic mechanism is governed mainly by hydroxyl radicals (•OH) and holes (h^+^).

**Figure 8:**
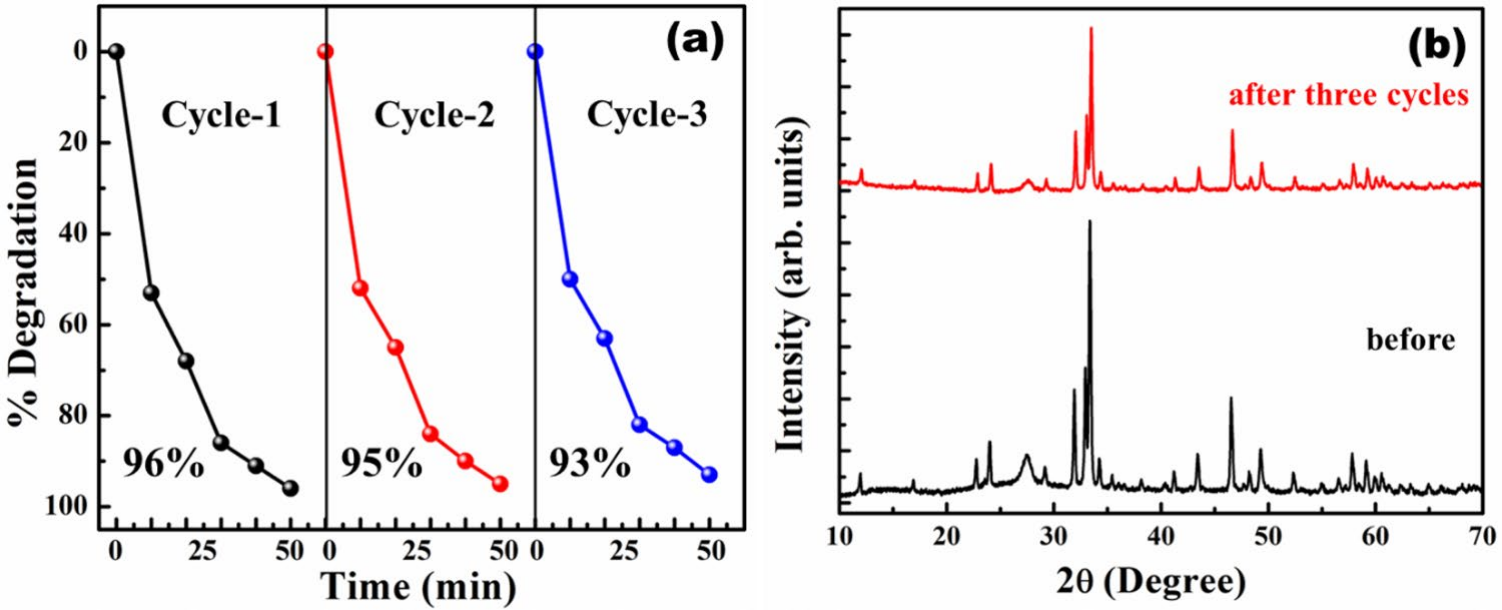
(**a**) The cyclic photocatalytic experiments for the degradation of MB using CCN50 (**b**) XRD patterns of CCN50 before, after the 3-cycles of photocatalytic degradation of MB.

Recycling and stability studies were conducted on g-C_3_N_4_/Ca_2_Fe_2_O_5_ (CCN50) heterostructure by degrading methylene blue (MB). The degradation efficiency of CCN50 was found to be reproducible up to three cycles (Fig. 8a). The slight reduction in efficiency in third cycle could be attributed to variation in sunlight intensity. No structural changes were observed in XRD pattern (Fig. 8b) of CCN50 post photodegradation. These studies revealed that as prepared heterostructures are stable and recyclable for practical usage.

The samples CFO, g-C_3_N_4_ and optimized composite CCN50 were further utilized for degrading Bisphenol-A (BPA). BPA is widely used polycarbonate plasticizer to manufacture plastic containers to pack and carry food items^50^ and was declared hazardous to human health, causing cancer, infertility, diabetes, and obesity etc.^51,52^.

**Figure 9:**
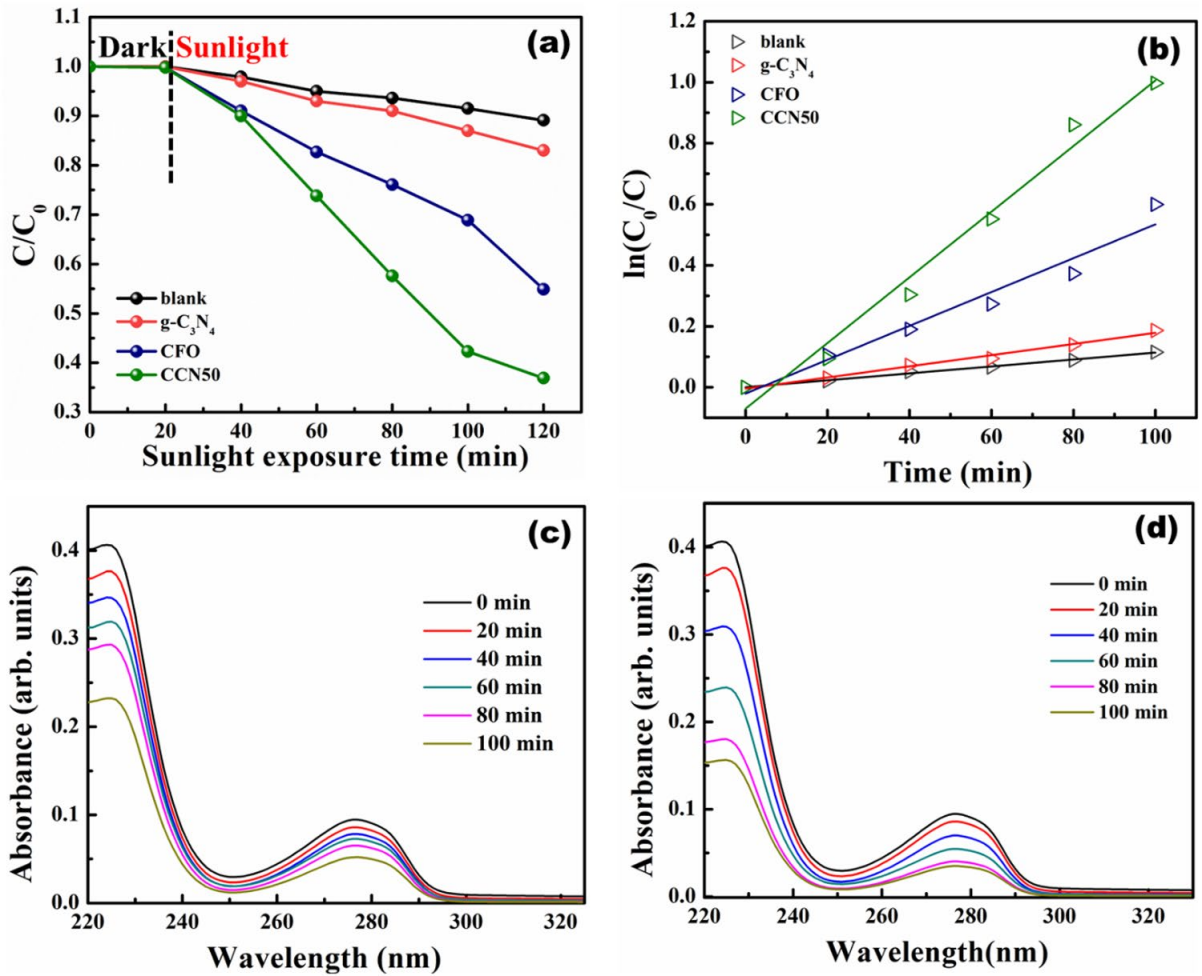
(**a**) Photocatalytic performance of CFO, g-C_3_N_4_, and CCN50 during degradation of BPA (**b**) corresponding reaction kinetics, and photodegradation profile of BPA using (**a**) CFO (**b**) CCN50.

In the present work, BPA degradation was carried out under natural sunlight. The percentage of degradation, first order rate constant plots and degradation profile of BPA using CFO and CCN50 are shown in Fig. 9(a-d). BPA could be degraded up to 45.1%, 17% and 63.1% with a rate constant 5.55 × 10^–3^ min^-1^, 1.83 × 10^–3^ min^-1^ and 10.76 × 10^–3^ min^-1^ using CFO, g-C_3_N_4_ and CCN50 respectively. In the present study too, CCN50 showed better photodegradation performance as compared to CFO and g-C_3_N_4_. The photocatalytic performance of g-C_3_N_4_/CFO heterostructures were compared and tabulated in table S1.

Possible degradation pathway for BPA is shown in figure S1. CFO possesses holes (h^+^) and hydroxyl radicals as active species for photocatalytic degradation^35^. These active species attack the quaternary carbon in BPA and form 2,2-bis(4-hydroxyphenyl)-1-propanol in Stage-I. The intermediate product further photocleaves into 4-hydroxybenzoate and 4-hydroxyacetophenone via C–C scission reaction in stage-II. These two intermediate products transform into aromatic formic and acetic acids in stage-III which further mineralizes into CO_2_, H_2_O and other degradation products in the final stage^53^. This process will continue until BPA degrades completely. The above studies reveal that g-C_3_N_4_/CFO composites are promising catalysts for degrading MB and BPA.

**Figure 10:**
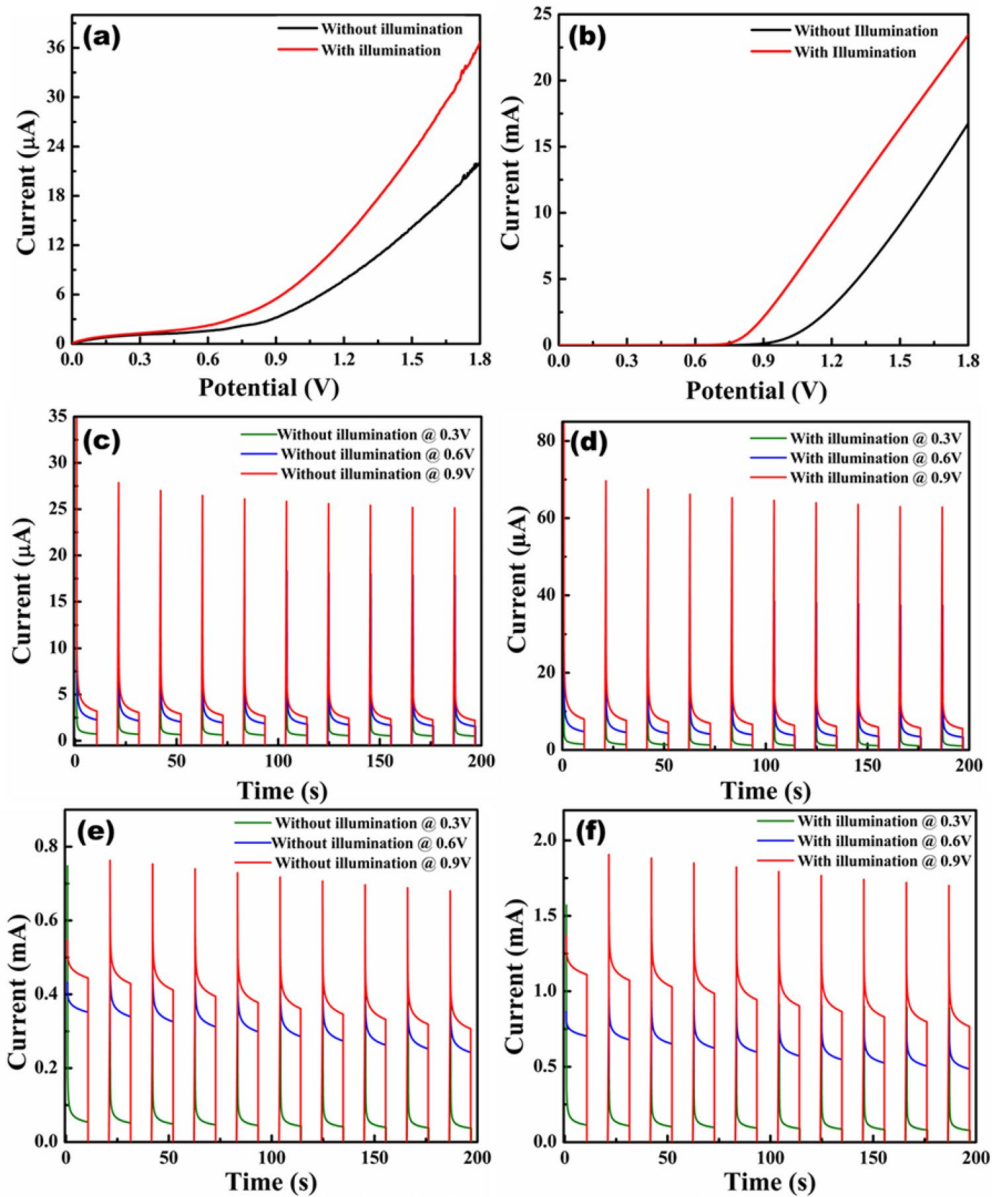
PEC characteristics of pure CFO and g-C_3_N_4_/CFO (CCN50) in 0.5 M KOH electrolyte under 100 mW/cm^2^ light intensity (**a** and **b**) LSV curves of CFO and CCN50 with and without light. (**c** and **d**) CA plots of CFO with and without light. (**e** and **f**) CA plots of CCN50 with and without light.

In order to examine the PEC properties of CFO and g-C_3_N_4_/CFO (CCN50) heterojunction, Linear sweep voltammetry (LSV) and Chronoamperometry (CA) studies were carried out with and without light illumination. From the LSV plots [Fig. 10(a & b)],it is clear that CCN50 electrode exhibits better photo response over pure CFO due to efficient charge separation and faster electron–hole transfer through Type-II heterojunction^54^. The photocurrents corresponding to pure CFO and CCN50 were observed from CA studies [Fig. 10(c-f)] at various potentials (0.3 V, 0.6 V and 0.9 V). The photocurrent for CCN50 was observed around 1.9 mA under constant illumination of light and for pure CFO electrode it was observed around 67.4 μA at 0.9 V. The photocurrent density improved remarkably in CCN50 heterojunction. The PEC studies revealed good photo response as well as efficient charge separation features of g-C_3_N_4_/CFO heterojunction over pure CFO. The photocatalytic and PEC studies reveal promising photoactivity of g-C_3_N_4_/CFO heterostructures as photoactive materials for solar energy harvesting applications.

## Conclusion

Brownmillerite CFO and g-C_3_N_4_ heterostructures are developed using simple solid-state route. Structural, microstructural and optical properties were analyzed using SEM, EDS, XPS and UV–Visible spectroscopy. Photocatalytic performance of as developed heterostructures were tested and compared by degrading organic effluents MB and BPA under sunlight. g-C_3_N_4_/CFO heterostructures show degradation efficiencies ~ 95.4% and ~ 63.1% for degrading MB and BPA respectively. The photoelectrochemical studies revealed higher photocurrents in g-C_3_N_4_/CFO heterostructures over CFO. The enhanced photodegradation efficiency was observed for g-C_3_N_4_/CFO heterostructures over bare Ca_2_Fe_2_O_5_ and g-C_3_N_4_. The photoelectrochemical studies revealed higher photocurrents in g-C_3_N_4_/CFO heterostructures over CFO, which  was attributed to suppression of electron–hole pair recombination. The systematic studies on these heterostructures revealed future potential of these newly developed heterostructures to be used for energy and environmental applications.

## Supplementary Information

Supplementary Information.
