# Supplementary material for: g-C3N4/Ca2Fe2O5 heterostructures for enhanced photocatalytic degradation of organic effluents under sunlight
Source: Sci Rep. 2021 Oct 4;11:19639. doi: 10.1038/s41598-021-99020-6 (PMC8490349; doi:10.1038/s41598-021-99020-6)
Supplement: Supplementary file 1 — Supplementary Information. [file 41598_2021_99020_MOESM1_ESM.docx]

**Supplementary Information**

**g-C_3_N_4_/Ca_2_Fe_2_O_5_ heterostructures for enhanced photocatalytic degradation of organic effluents under sunlight**

Durga Sankar Vavilapalli^1^, Raja Gopal Peri^2^, R. K. Sharma^3^, U. K. Goutham^3^, Muthuraaman B^2^, M. S. Ramachandra Rao^4^, Shubra Singh^1,*^

*^1^Crystal Growth Centre, Anna University, Chennai-600025, India.
^2^Department of Energy, University of Madras, Chennai-600025, India.
^3^Technical Physics Division, Bhabha Atomic Research Centre, Mumbai - 400085, India.
^4^Nano Functional Materials Technology Centre and Department of Physics, Indian Institute of Technology Madras, Chennai-600036, India.*

*^*^Corresponding author’s email: shubra6@gmail.com*


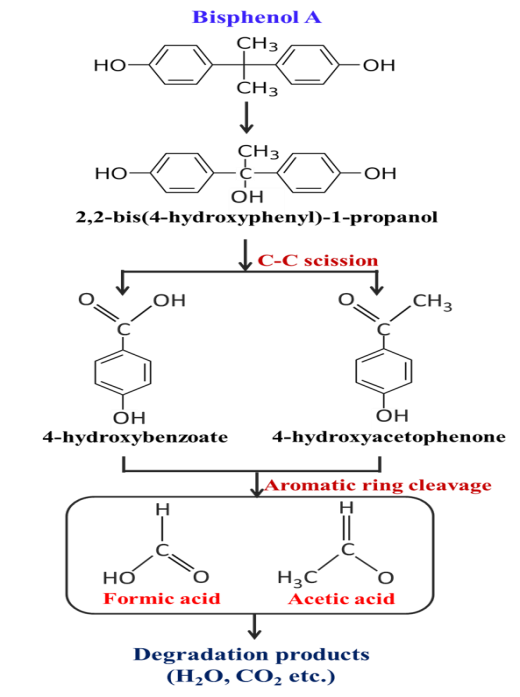


**Figure S1:** Possible photodegrading pathway of Bisphenol-A.

**Table S1:** A comparison of the photocatalytic performance of g-C_3_N_4_/CFO for degradation of BPA with other existed photocatalysts in visible light.

| **S.No** | **Catalyst** | **Dye concentration and catalyst loading** | **%degradation and degradation time** | **Light source** | **Ref.** |
| --- | --- | --- | --- | --- | --- |
| 1 | CFO g-C_3_N_4_/CFO (CCN50) | BPA (50mg/L), 50mg/L | 45.1% (100 min) 63.1% (100 min) | Sun light | This work |
| 2 | TiO_2_ | BPA (10mg/L), 20mg/30ml | 10% (120 min) | Vis. light | ^51^ |
| 3 | N doped TiO_2_ | BPA (10mg/L), 50mg/30ml | 13% (180 min) | Vis. light | ^52^ |
| 4 | ZnO | BPA (30mg/L), 40mg/50ml | 29.30% (8h) | Sun light | ^53^ |
| 5 | FDU-PdPcS | BPA (60mg/L), 20mg/L | 56% (6h) | Sun light | ^54^ |
| 6 | BiFeO_3_ BiOI | BPA (1x10^-5^M), 75mg/75ml | 7% (120 min) 36% (120 min) | Vis. light | ^55^ |
| 7 | PbBiO_2_Br | BPA (10mg/L), 50mg/50ml | 38.4% (120 min) | Vis. light | ^56^ |
| 8 | Bi_2_MoO_6_ | BPA (10mg/L), 100mg/100ml | ~40% (260 min) | Vis. light | ^57^ |
| 9 | N-doped BiFeO_3_ | BPA (30mg/L), 35mg/70ml | 24% (120 min) | Vis. light | ^58^ |
| 10 | BiOCl | BPA (10mg/L), 10mg/40ml | 20% (120 min) | Vis. light | ^51^ |
